# Sexual Differences in Appendages of a Fossorial Narrow-Mouth Frog, *Kaloula rugifera* (Anura, Microhylidae)

**DOI:** 10.3390/ani15172566

**Published:** 2025-08-31

**Authors:** Wenyi Zhang, Xianzheng Wang, Jin Huang, Xiuping Wang, Bin Wang, Jianping Jiang, Bingjun Dong, Meihua Zhang

**Affiliations:** 1 College of Life Sciences, Shenyang Normal University, Shenyang 110034, China; zhangwy0523@163.com (W.Z.); wxiuping0523@163.com (X.W.); 2 Chengdu Institute of Biology, Chinese Academy of Sciences, Chengdu 610213, China; wangbin@cib.ac.cn (B.W.); jiangjp@cib.ac.cn (J.J.); 3 Shengda Hydropower Co., Ltd., Sinohydro Group Ltd., Leshan 614000, China; scslhwangxzh@powerchina.cn (X.W.); sczssdhuangjin@powerchina.cn (J.H.)

**Keywords:** geometric morphometrics, appendicular skeletons, sexual dimorphism

## Abstract

Skeletons serve as the body’s fundamental structure and perform multiple vital functions, and thus even subtle modifications can lead to functional changes. However, studies on sexual shape dimorphism in the appendicular skeletons of anurans remain scarce, especially for fossorial species. Herein, we investigated the sexual differences in the external morphology and internal skeletal shape of girdles and limbs of fossorial *Kaloula rugifera* during the breeding season. The results reveal the following: (1) Males exhibit significantly longer forelimbs and hindlimbs, suggesting advantages in male—male competition and amplexus. (2) Males possess more curved and dilated coracoids and broader pubis-ischium regions, which may enhance forelimb contraction efficiency and hindlimb locomotor performance. (3) Males feature a more developed crista ventralis, likely enhancing forelimb flexion for tightly grasping females during mating. In contrast, females display a torsioned femur and robust hindlimb morphology, suggesting adaptations for digging and load-bearing. These findings provide insights into how anurans optimize reproductive fitness and ecological adaptations through morphological specialization. To our knowledge, this study presents the first examination of sexual shape dimorphism in anuran appendicular skeletons utilizing three-dimensional geometric morphometrics.

## Simple Summary

The appendicular skeletons are of great importance in vertebrate adaptation and evolution. However, the research on sexual differences mainly focuses on the external morphological traits and muscle mass of appendages. This study presents the first comprehensive and detailed investigations into the appendicular skeletons of a fossorial anuran species using 3D geometric morphometrics. The results show that there are obvious sexual shape differences in the coracoids, pubis-ischium regions, and humerus. These findings facilitate our understanding of sex-specific behaviors, reproductive strategies, and ecological adaptations.

## 1. Introduction

Skeletons, the fundamental structures of vertebrates, perform multiple vital functions, including protecting internal organs, anchoring muscles and tendons, and participating in locomotion [1,2]. Due to these essential roles, even subtle structural variation in the skeleton can lead to dramatic functional changes [3]. Although vertebrate skeletons exhibit considerable phylogenetic conservation overall [4], they are also influenced by genetic variation and environmental pressure that generate morphological diversity. A common and intriguing manifestation of such variation is sexual dimorphism in the skeletal morphology, which occurs across vertebrates and exhibits differences in the size, shape, and proportions between males and females [5]. Examples range from the robust antlers of male deer [6] to the dimorphic skull in lizards [7] and frogs [8], the apparent larger scapulae in male elephants [5], and the wider pelvic girdle in female humans [9]. These dimorphic traits often reflect sex-specific selective pressures that facilitate adaptation to divergent survival, reproduction, and life-history strategies [10]. Among tetrapods, sex-related skeletal variation is most pronounced in the postcranial skeletons [11].

The appendicular skeletons are of great importance in vertebrate adaptation and evolution. Their emergence provides the essential biomechanical foundation for body weight support and land locomotion, representing a key innovation in vertebrate terrestrialization [12,13]. Sexual dimorphism in the appendicular skeleton occurs across vertebrate taxa and follows general patterns in certain structures. For instance, males generally exhibit more robust forelimb skeletons, a morphology associated with improved locomotor performance and competitive ability [14]. Females typically possess broader pelvic girdles, an adaptation linked to functional demands such as pregnancy, birth, and load-bearing [11]. Studying sexual dimorphism in the vertebrate appendicular skeletons will provide critical insights into underlying evolutionary mechanisms, given their primary role in locomotion and behavior.

Anurans display peculiar appendicular morphological diversity to meet the demands of diverse locomotor abilities [15,16,17], microhabitat utilization [18,19], and reproductive strategies [20,21], which makes them ideal models to investigate how selective pressures shape skeletal dimorphism. The pelvic girdles and hindlimbs are critical determinants of locomotor performance in anurans [18,22,23,24]. For example, the inter-ilial width negatively correlates with locomotor efficiency [25,26]. The hindlimb length serves as a reliable predictor of locomotor types [27]. The anurans with relatively longer hindlimbs tend to be good jumpers [28], while anurans with shorter hindlimbs and their elements tend to be walkers, hoppers, or burrowers [29,30]. Additionally, terrestrial and arboreal jumpers possess an enlarged omosternum, potentially enlarging the origin area of the coracoradialis muscle, which plays a critical role in shock absorption upon landing [19,31]. Fossorial species tend to have thicker and shorter limbs to support moving substrates and progressing underground [32,33]. Furthermore, the forward-burrowing species tend to have more developed crista ventralis in the humerus [32].

Anurans exhibit pronounced sex-specific behaviors during reproductive activities, creating distinct biomechanical demands on their appendicular skeletons. These functional divergences likely drive selective pressures toward skeletal dimorphism [21]. A striking example is the well-developed crista ventralis, a humeral adaptation that optimizes forelimb biomechanics during amplexus [34,35]. To the best of our knowledge, the current research on sexual dimorphism in anuran limb skeletons has primarily relied on linear measurements, with surprisingly limited investigation into three-dimensional shape variation [20,21]. Furthermore, studies examining sexual differences in anuran girdles remain rather scarce, especially for the pectoral girdles. Landmark-based geometric morphometrics offer a powerful approach for detecting subtle shape variations at both intra- and interspecific levels [36,37]. This method quantifies biological geometry using discrete anatomical landmarks and semi-landmarks and homologous points representing key morphological features that are reliably identifiable across specimens [38,39]. By transforming morphological structures into spatially coordinated point configurations, shape differences can be visualized and analyzed quantitatively. Applying geometric morphometrics to sexual shape variation could yield novel insights into functional adaptations of skeletal structures [36,40].

Microhylidae, comprising 764 recognized species, is the third largest anuran group with a global distribution. This family exhibits remarkable morphological and ecological variation, including terrestrial, arboreal, and fossorial adaptations [41,42]. Among these ecotypes, fossorial species exhibit particularly pronounced diversification in their external morphology, osteology, and myology [17,32,43,44]. However, investigations of sexual dimorphism in fossorial species remain limited, primarily due to the challenges of observing these frogs during non-breeding periods [45]. This knowledge gap hinders our understanding of their potential sex-specific morphological adaptations.

*Kaloula rugifera* (Anura, Microhylidae), a typical hindlimb-burrowing anuran species, is only distributed in Sichuan Province and the southernmost Gansu Province of China [46]. It exhibits a pronounced phenotypic dimorphism, with males possessing vocal sacs and linea musculina [46,47,48]. Its reproduction occurs after heavy rains during the breeding season, and it often hides in soil holes or rock crevices during the non-breeding season [48]. To date, investigations on this species have been focused on the karyotype [49], phylogenetic relationship [50], reproductive ecology [47,48], and advertisement call [51]. However, the differences in appendages between the sexes have not yet been examined. In this study, we aim to (1) comprehensively document the sexual differences in the appendages in the external morphology and internal skeletons and (2) elucidate the functional associations between these morphological differences and reproductive behaviors as well as ecological adaptations. This study provides novel insights into the behavioral and reproductive strategies of this species and also provides fundamental data to elucidate how selective pressures differentially shape skeletal dimorphism across vertebrates.

## 2. Materials and Methods

### 2.1. Sampling

A total of 36 specimens (18 males and 18 females) of *K. rugifera* were collected by hand on the evening of 17 and 23 July 2024 from Longquanyi (30°34′ N, 104°17′ E, 522.79 m), Chengdu City, Sichuan Province, China. Sex determination was based on external morphological characteristics. Specifically, males possess two distinct white linear musculina, thickened ventral glands, and darkened vocal sacs, while females lack these features [46]. Subsequently, these specimens were transferred to the laboratory, and then, they were sacrificed by immersion in 5 g/L MS-222 (tricaine methanesulphonate) solution [52,53].

### 2.2. External Morphometrics Measurements

**Figure 1:**
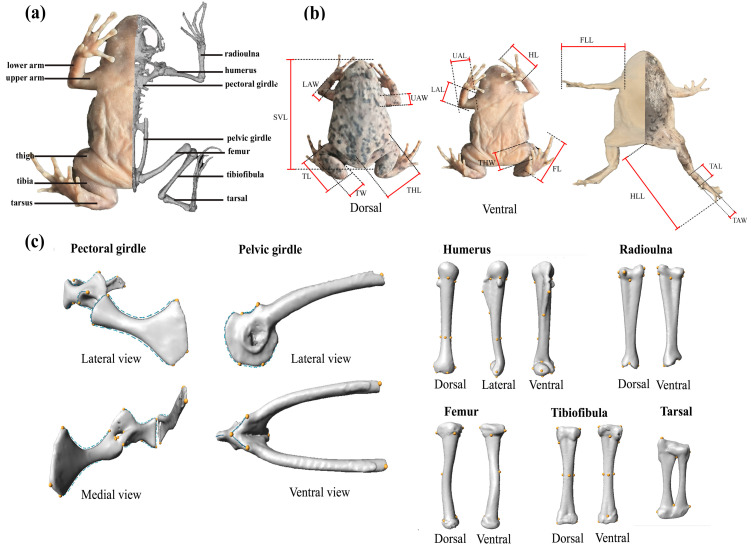
Protocol for external morphometric measurements and three-dimensional landmark-based geometric morphometrics of the appendicular skeletons used in this study. (**a**) External morphology and the three-dimensional reconstructed skeletal model of *Kaloula rugifera*; (**b**) details for linear morphometric measurements; and (**c**) three-dimensional landmarks and semi-landmarks of appendicular skeletons. Yellow points represent landmarks; blue points represent semi-landmarks; and blue arrows in the pectoral girdle and pelvic girdle represent the directions of semi-landmark curves.

Fifteen morphological traits were measured following Fei et al. [46] and Watters et al. [54] with a digital vernier caliper (DEGUQMNT MNT-200, Shanghai Meinaite Industrial Co., Ltd., Shanghai, China) to the nearest 0.01 mm (Figure 1a,b). These measurements included snout–vent length (SVL), upper arm length (UAL), upper arm width (UAW), lower arm length (LAL), lower arm width (LAW), hand length (HL), forelimb length (FLL), thigh length (THL), thigh width (THW), tibia length (TL), tibia width (TW), tarsus length (TAL), tarsus width (TAW), foot length (FL), and hindlimb length (HLL). Additionally, the body mass of each individual was measured to the nearest 0.01 g with an electronic balance (Gason GS-100, Shenzhen Haotian Network Technology Co., Ltd., Shenzhen, China). To account for body size, all measurements were standardized to SVL for further analysis (Table S1).

### 2.3. MicroCT Scanning

Skeletal shape was captured using a high-resolution X-ray scanner (Quantum GX microCT Imaging System, PerkinElmer^®^, Waltham, MA, USA) at Chengdu Institute of Biology, Chinese Academy of Sciences. Scanning parameters were set at 90 kV, 88 μA, 14 min, and 512 projections [55]. The resulting images were segmented and reconstructed into 3D digital models (STL files) using Mimics v. 21.0 (Materialise^®^ HQ Technologielaan, Leuven, Belgium). Finally, the reconstructed models were adjusted and exported as PLY files using MeshLab v. 2023.12 (ISTI-CNR, Pisa, Italy) for further analysis.

### 2.4. Geometric Morphometrics Landmarking

Except for the pelvic girdle, the remaining appendicular skeletons were marked on the right side to reflect their characteristics. Landmarks and semi-landmarks were digitized on the 3D models using Checkpoint v. 2020 (Stratovan Corporation, Sacramento, CA, USA). Landmark configurations were based on homologous points following previous studies [17,18,32,56]. Specifically, for the pectoral girdle, 21 landmarks and 245 semi-landmarks were used; for the humerus, 17 landmarks; for the radioulna, 10 landmarks; for the pelvic girdle, 8 landmarks and 88 semi-landmarks, and for the femur, tibiofibula, and tarsal, 6, 12, and 6 landmarks were used, respectively (Figure 1c). All landmarkings were performed by W. Z. to minimize inter-observer error.

### 2.5. Statistical Analyses

For external morphometric data, first, normality and variance homogeneity were, respectively, verified through Shapiro–Wilk and Levene’s tests. Results showed all data were normally distributed and homogeneous (*p* > 0.05) except for variance homogeneity of body mass (*p* < 0.05). Second, Welch’s *t*-test was employed to compare body mass differences between sexes, while the remaining traits were compared using the independent *t*-test. Third, linear regression analysis was performed on sexually dimorphic traits, regarding SVL as the independent variable. All above analyses were conducted using SPSS v. 27.0 (IBM Corp., Chicago, IL, USA), with statistical significance set at *p* < 0.05. Fourth, to characterize the overall morphological patterns between sexes, principal component analysis (PCA) was performed and visualized using the R package stats v. 4.4.1 and ggplot2 v. 3.5.1 in R v. 4.3.3. The Kaiser Rule criterion was used to determine the number of factors extracted, and it only retained factors that had Eigenvalues greater than 1.

For geometric morphometric data, first, all landmark configurations were aligned using generalized Procrustes analysis (GPA) to remove effects in translation, rotation, and scale. The resulting Procrustes coordinates represented the shape of the skeleton, while the centroid size quantified the size of skeleton. Second, to explore and visualize skeleton shape similarity and variability between sexes, we performed principal component analysis (PCA) and generated Thin-Plate Spline (TPS) deformation grids using the functions “gm.prcomp” and “plotRefToTarget”. Third, Procrustes ANCOVA was used to quantify the sex-related differences in shape, regarding sex as the dependent variable, with 9999 permutations using the function “procD.lm”. Fourth, we regressed log-transformed centroid size against shape to examine allometric patterns integrally and separately for sexes using “procD.lm”. Distinct allometric results were visualized using regression scores and fitted scores to summarize the multivariate regression. All geometric morphometrics analyses were performed using the R package geomorph v. 4.0.10 [57].

## 3. Results

### 3.1. Comparisons of External Measurements

The female *K. rugifera* exhibited a significantly greater SVL and body mass (*p* < 0.001; Table 1). After accounting for the SVL, independent *t*-tests demonstrated significant sexual dimorphism in 9 out of 14 external limb measurements. Specifically, males showed significantly longer upper arms, lower arms, complete forelimbs, tibiae, tarsi, feet, and complete hindlimbs than females (*p* < 0.01; Table 1). Additionally, males displayed significantly greater widths in both the upper arm and lower arm than females (*p* < 0.05; Table 1).

**Figure 2:**
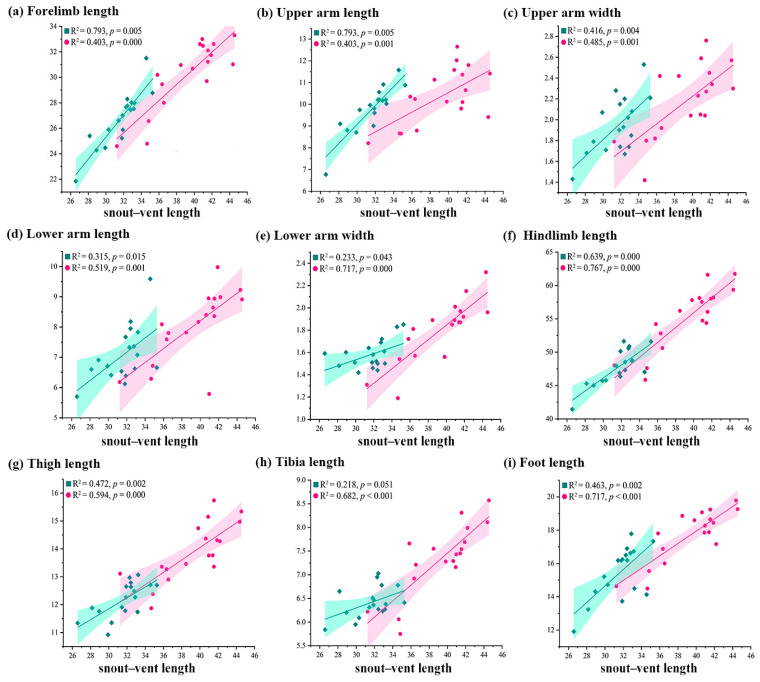
Linear regressions of external limb morphology against snout–vent length for males (green rhombus symbols) and females (pink circular symbols) of *Kaloula rugifera*. These external morphological traits are significantly different between sexes.

Allometric analyses revealed sex-specific growth patterns: males showed significantly greater coefficients for the forelimb length, upper arm length, upper arm width, lower arm length, and foot length relative to the SVL than females (Figure 2a–d,i). In contrast, females exhibited significantly greater allometric coefficients than males for the lower arm width, hindlimb length, thigh length, and tibia length relative to the SVL (Figure 2e–h).

**Figure 3:**
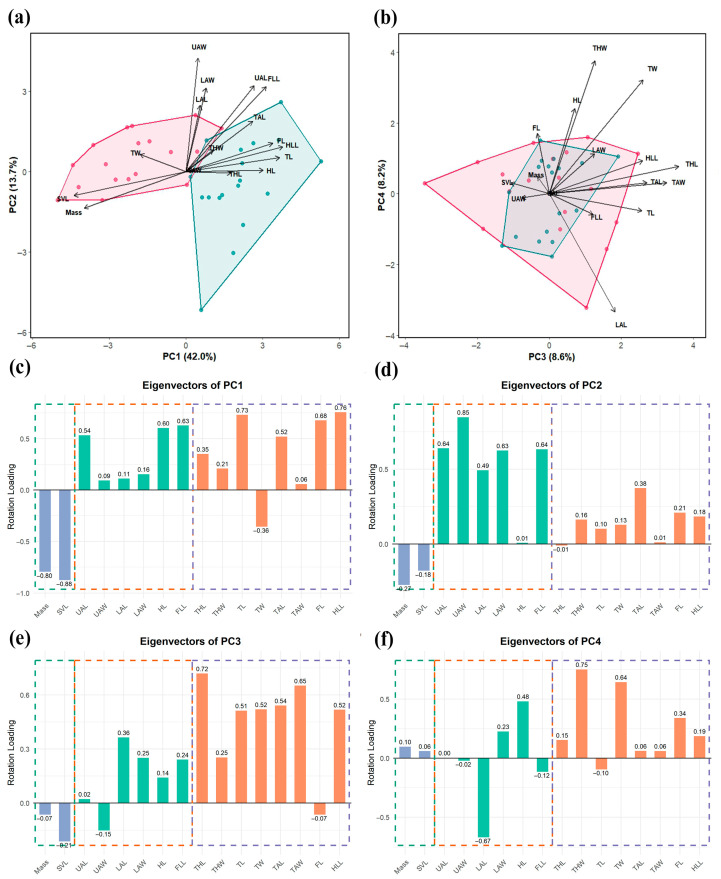
Principal component analysis of the 16 external measurements. (**a**,**b**) Plot of first four principal components of morphometric data, with males indicated in green, females in pink. (**c**–**f**) Eigenloadings on PC1–PC4.

Four principal components with Eigenvalues greater than one were extracted and accounted for a total variance of 72.5%. PC1 accounted for 42.0% of the total variance and effectively separated the males from the females based on external morphological measurements, whereas the remaining components (PC2–PC4) failed to demonstrate clear sexual morphological traits (Figure 3a,b). The five variables with the higher loadings were the snout–vent length (SVL, −0.88), body mass (−0.80), hindlimb length (HLL, 0.76), tibia length (TL, 0.73), and foot length (FL, 0.68) (Figure 3c). This pattern suggested that PC1 primarily represented an axis of size-related variation and locomotor adaptation, with positive loadings indicating limb length dimorphism and negative loading (SVL and body mass), potentially reflecting body size dimorphism. Variables in PC2 with the higher loadings were the upper arm width (UAW, 0.85) and upper arm length (UAL, 0.64), which accounted for 13.7% of the total variation (Figure 3a,d). Females exhibited constrained morphological variation clustered exclusively in the positive direction (Figure 3a). PC3 and PC4 collectively accounted for 16.8% of the total variation, primarily associated with the hindlimb morphology, with the highest loadings being the thigh length (THL, 0.72) and thigh width (THW, 0.75) (Figure 3e,f). Notably, both sexes showed an overlap in this morphospace (Figure 3b).

### 3.2. Comparisons of Girdle and Limb Shape

**Figure 4:**
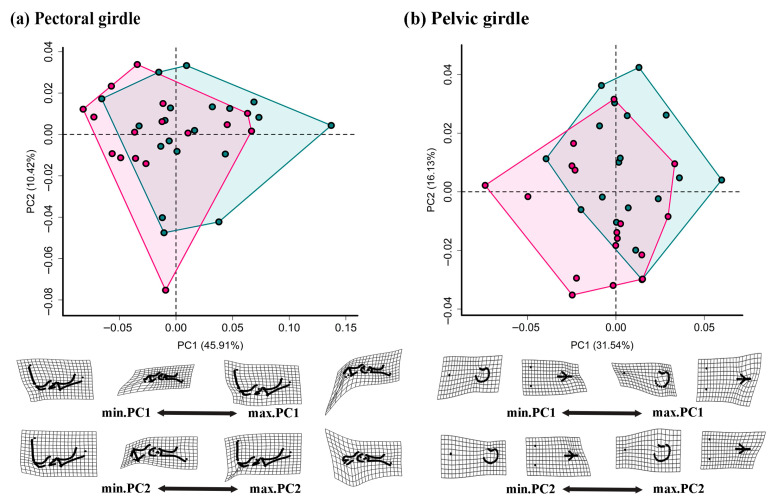
Morphospaces and deformation grids of the girdle skeletons. Two pairs of shape meshes associated with the PC1 and PC2 represent the differences between specimens at the two ends of the corresponding shape axes (PC1 and PC2). Specifically, deformation grids showing shape change from the average (0, 0) to the negative (min) and positive (max) values of PC1 and PC2. Color corresponds to gender type, with males indicated in green and females in pink.

For pectoral and pelvic girdles, the first three PCs cumulatively explained 64.32% and 56.06% of the total shape variation, respectively (Figure 4 and Figure S1a,b). Although a morphospace overlap occurred, PC1 notably contributed to separating samples by sex, with males mainly clustering in the positive region. Moreover, PC3 introduced additional nuances in the morphological variation. Specifically, for the pectoral girdle, males were characterized by a longer, wider, and more curved coracoid, particularly in the medial region. In contrast, females had a longer scapula and cleithrum, as well as a broader distal scapula. For the pelvic girdle, males exhibited a wider pubis and ischium, whereas females had a longer ilium and a wider dorsal condyle.

**Figure 5:**
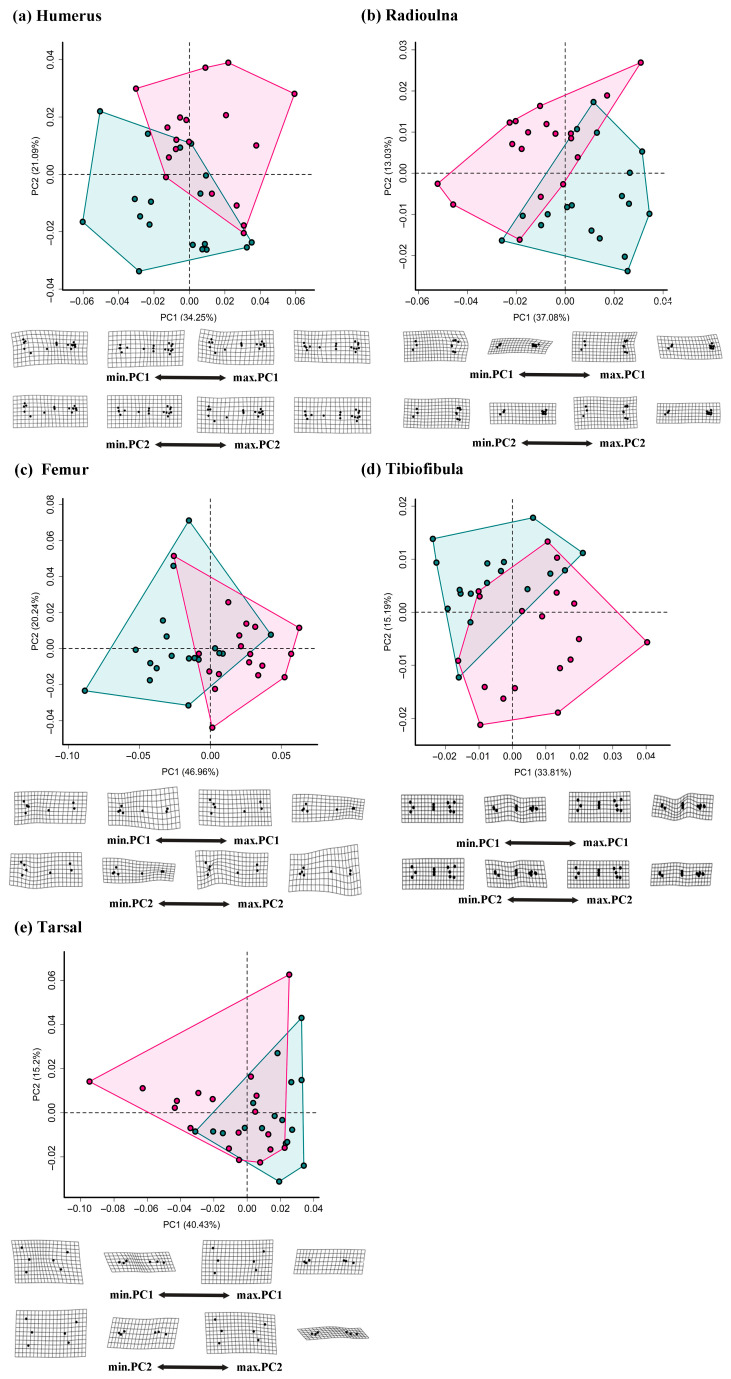
Morphospaces and deformation grids of the limb skeletons. Two pairs of shape meshes associated with the PC1 and PC2 represent the differences between specimens at the two ends of the corresponding shape axes (PC1 and PC2). Specifically, deformation grids showing shape change from the average (0, 0) to the negative (min) and positive (max) values of PC1 and PC2. Color corresponds to gender type, with males indicated in green and females in pink.

For the humerus and radioulna, the first three PCs cumulatively explained 64.59% and 59.52% of the total shape variation, respectively (Figure 5a,b and Figure S1c,d), while PC1 effectively separated males and females. Specifically, males exhibited a wider humeral neck and a more developed crista ventralis, while females had a smaller neck and crista ventralis but a slightly extended distal condyle. Moreover, males showed a longer radioulna diaphysis, particularly in the proximal region, and a more vertical sesamoid on the lateral side of the distal radius.

For the femur, tibiofibula, and tarsal, the first three PCs cumulatively explained 60.84%, 69.62%, and 76.98% of the total shape variation, respectively (Figure 5c–e and Figure S1e–g). For the femur, females exhibited greater femoral torsion and thicker diaphysis along the positive PC1 compared to males. For the tibiofibula, both PC1 and PC2 reflected width variation, with males having a thinner diaphysis than females. In the tarsal, males clustered predominantly in positive PC1 regions, corresponding to a longer tarsal foramen than females. Conversely, females displayed a more robust bone morphology at both ends and greater relative height differences between tarsal foramen extremities.

**Figure 6:**
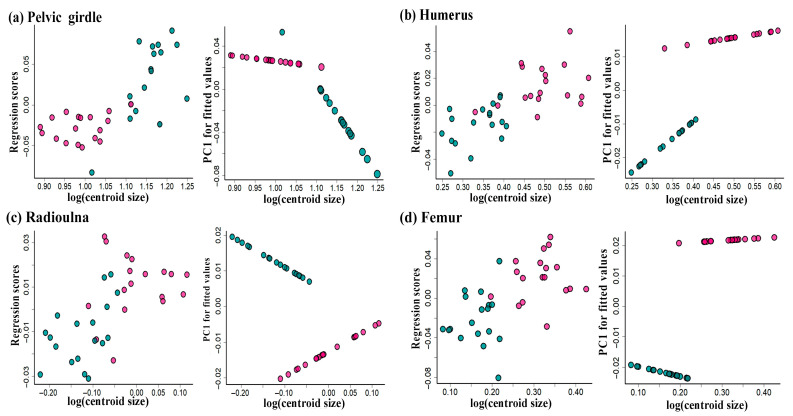
Plots of allometric variations as well as regression score vs. skeleton size (log-transformed centroid size). Color corresponds to gender type, with males indicated in green and females in pink.

The Procrustes ANOVA revealed significant sexual shape dimorphism in the pelvic girdle, humerus, radioulna, femur, tibiofibula, and tarsal (*p* < 0.05) but not in the pectoral girdle (*p* > 0.05; Table 2). However, the skeleton size only differed in the pelvic girdle, humerus, and tarsal between males and females (*p* < 0.05; Table 2). Multiple ANCOVA regressions indicated that the centroid size significantly influenced the shape of the pelvic girdle, humerus, radioulna, and tarsal shapes (*p* < 0.05), while sexually dimorphic allometric trajectories were exhibited in the pelvic girdle, humerus, and femur (*p* < 0.05; Table 2; Figure 6). This indicated that, within the studied samples, at least a subset of males and females exhibit distinct skeletal shapes, as suggested by the different allometric slopes.

## 4. Discussion

### 4.1. Sexual Size Dimorphism in External Morphology

Our results reveal remarkable sexual dimorphism in the external morphology of *K. rugifera*. Consistent with the female-biased size dimorphism widely documented in anurans, females of *K. rugifera* exhibit significantly greater body length and weight than males, which may be closely linked to their reproductive investment strategies [58]. This divergence may stem from differences in the growth rate and age at sexual maturity between sexes, with females typically exhibiting reduced growth rates and/or delayed maturation [59,60]. The prolonged growth period enables females to achieve larger sizes, because the body size is a critical constraining factor for the reproductive capacity, with this reproductive advantage being most pronounced in explosive-breeding and miniaturized species [61]. For *K. rugifera*, the female-biased body size most likely increases the reproductive output to ensure breeding success under explosive breeding constraints [41,62]. Except for reproduction, body mass as a fundamental parameter in ecology has positive relation with the metabolic rates and energy intake [63]. For explosive-breeding species, including *K. rugifera*, the larger mass enables increased energy reserves, thereby meet the high energy demands of oogenesis and oviposition [63]. Additionally, this dimorphism may be driven by differential predation pressure, as adult males are often more exposed and face higher risks during breeding events, and their smaller size could reduce detectability by predators [64].

After accounting for the SVL, males of *K. rugifera* have significantly longer forelimbs (upper arms and lower arms) and hindlimbs (tibiae, tarsi, and feet), and exhibit greater growth rates in the lengths of the upper arms, lower arms, forelimbs, and feet. These male-biased patterns indicate that sexual selection may act as the strong driving force, because the limb sizes of males play important roles in the anuran mating [65]. Specifically, males with longer and wider forelimbs may have enhanced grasping ability during amplexus [66,67], and resist displacement from unpaired males [7], which is a crucial behavior directly linked to reproductive success. The hindlimb length is usually correlated with the locomotor ability, efficiency, and dispersal distance in anurans [28,68]. For *K. rugifera*, males with longer hindlimbs likely possess enhanced propulsive force, enabling them to pursue and mate with females more efficiently and quickly in the breeding water bodies [48,65,69]. For explosive-breeding species like *K. rugifera*, the ability to rapidly locate and clasp a female is crucial, as the rewards of successful mating far outweigh the potential costs of errors [69]. Additionally, as mentioned above, males face higher predation pressure than females, especially when calling to attract mates [70]. Stronger locomotor ability in this case may adapt to effectively reduce the costs associated with predator avoidance [23,71,72]. Moreover, longer hindlimbs in males are closely associated with an increased competitive ability during amplexus [20], which help them expel other rival males and secure their position during mating [21]. These findings suggest that sexual selection may play an important role in shaping the sexual limb dimorphism in *K. rugifera*, favoring traits that enhance both locomotor performance and competitive success.

### 4.2. Sexual Shape Dimorphism in Appendicular Skeletons

Behaviors are accomplished by the forces generated from the contraction of muscles and tendons attached to skeletons [73]. Subtle differences in skeletal morphology may alter the origin and insertion of muscles and thus change their moment arms [74]. In anurans, pectoral girdles and forelimbs function synergistically in transferring and absorbing landing impacts, as well as other behaviors such as locomotion and amplexus [12,16,21,34]. The males of *K. rugifera* have elongated, wider, and curved coracoids, which are distinct from females (Figure S2). This specific configuration of the coracoid might change the origin of the muscles (e.g., M. pectoralis epicoracoidea and M. coracoradialis) dorsally and posteriorly, which adapts to improve the forelimb retraction effectiveness by increasing its moment arm [22,75]. Meanwhile, males of *K. rugifera* exhibit a more developed humerus crista ventralis, which has also been found in *Quasipaa robertingeri* (Anura, Dicroglossidae) [35]. The prolongation of the coracoid and the enlarged crista ventralis potentially enlarge the origin and insertion regions of the muscles, such as M. coracoradialis and M. deltoideus, which are pivotal in forearm flexion [7,21]. Both these modifications seem to be advantageous for the mechanical advantage of forelimbs to adapt to the amplexus behavior of males during mating [19,32]. Compared with slender skeletons, robust skeletons are typically associated with strong muscles and are better able to resist mechanical stress [56]. For females of *K. rugifera*, the thicker scapulas and coracoids may be responsed to the need for absorbing larger landing forces caused by a larger body mass [34,44,76].

The males of *K. rugifera* have wider pubes and ischia, which would accommodate enlarged attachment areas for muscles such as M. hemimembrane and M. obturator. These hindlimb flexors contribute to femur retraction and knee joint flexion, thereby, facilitating hindlimb propulsion during locomotion [77]. Additionally, compared with females, the narrower inter-ilial width in males may further enhance pelvic leverage and facilitate faster launch speeds and greater leap distances [25,26]. Such morphological specializations likely augment the muscle power output and locomotor frequency, ultimately providing advantages in male reproductive competition [18,40,78]. Females exhibit wider and larger ilia, which might be a consequence of fecundity selection to increase the carrying egg space and maximize the reproductive output [21]. For hindlimb skeletons, the observed femoral torsion is particularly fascinating, with females exhibiting a greater degree of torsion. Anurans, reptiles with a sprawling posture, and crouched mammals all exhibit pronounced femoral torsion, suggesting this may be an ancestral trait in tetrapod limb mechanics [79,80,81]. It has been reported that gravity can influence skeletal geometry [82], and different loadings may induce biological structure adaptive remodeling in shapes and microstructures [83]. The females of *K. rugifera* experience elevated gravitational loading (mass and egg-laying) and potentially increase femoral torsion as an adaptive response. This suggests that the substantial body mass disparity between sexes leads to the femoral morphological divergence. Additionally, diverse microhabitats and locomotor strategies impose distinct mechanical loads on bones [82], whereas locomotion often induces peak mechanical loads [79]. For hindlimb-digging anurans, including *K. rugifera*, the hindlimbs act as an external lever to generate force to excavate [84]. We hypothesize that the enhanced femoral torsion in females may correlate with stronger burrowing performance. The greater femoral torsion could probably enhance the mechanical advantage of hindlimb muscles by altering the line of action of the femur to improve the digging efficiency for females [32,85]. Interestingly, the femur, tibiofibula, and tarsal are thicker in females than in males, and developed bones are typically associated with robust muscles. This may indicate that females have greater hindlimb propulsive force to withstand the higher mechanical stresses associated with hindlimb digging [85]. It is likely that these modifications could avoid high daytime temperatures and periods of dryness or predator risk caused by large bodies [86,87]. Because the jumping ability of burrowing frogs decreases more rapidly with their body mass [88].

Identifying associations between skeletal shapes and functions is critical for determining how skeletons adapt to their biological roles [89]. The three-dimensional geometric morphometrics analyses reveal significant sexual dimorphism in the shape of the appendicular skeletons of *K. rugifera*. These findings provide insights into how anurans optimize fitness through morphological adaptations to meet reproductive and ecological demands.

## 5. Conclusions

This study provides the first document of sexual dimorphism in both the external limb morphology and internal appendicular skeletal shape and size in an anuran species. Males exhibit significantly longer limbs, and more robust skeletal elements than females, including more curved and dilated coracoids, wider pubis-ischium regions, and more developed crista ventralis. These morphological differences likely confer competitive and locomotor advantages, thereby improving reproductive success. In contrast, females exhibit more torsioned femurs and robust hindlimbs, which may reflect structural adaptations to hindlimb digging. These findings advance our understanding of how anurans optimize their fitness through sex-specific morphological adaptations to reproductive and ecological demands.

## Figures and Tables

**Table 1:** Differences in external morphological traits between males and females of *Kaloula rugifera*, with *p* values less than 0.05 given in bold.

Measurements	Levene’s Test		Mean ± Standard Deviation		t	*p*
	F	*p*	Male	Female		
Snout–vent length	6.495	0.016	31.661 ± 2.181	39.315 ± 3.644	7.647	**<0.001**
Body mass	17.362	<0.001	3.207 ± 0.684	6.801 ± 1.994	7.232	**<0.001**
External Morphology of Limbs (standardized to SVL)						
Upper arm length	3.092	0.088	0.309 ± 0.019	0.264 ± 0.025	−6.041	**<0.001**
Upper arm width	0.025	0.876	0.061 ± 0.007	0.055 ± 0.007	−2.706	**0.011**
Lower arm length	1.240	0.273	0.224 ± 0.023	0.204 ± 0.020	−2.724	**0.010**
Lower arm width	0.018	0.894	0.050 ± 0.004	0.046 ± 0.004	−3.188	**0.003**
Hand length	4.787	0.036	0.312 ± 0.016	0.302 ± 0.022	−1.533	0.135
Forelimb length	0.768	0.387	0.845 ± 0.030	0.771 ± 0.037	−6.590	**0.000**
Thigh length	0.344	0.561	0.434 ± 0.036	0.413 ± 0.026	−1.938	0.061
Thigh width	0.628	0.434	0.101 ± 0.008	0.098 ± 0.009	−0.972	0.338
Tibia length	0.009	0.924	0.386 ± 0.020	0.355 ± 0.023	−4.330	**<0.001**
Tibia width	0.004	0.948	0.075 ± 0.009	0.079 ± 0.009	1.063	0.295
Tarsus length	0.126	0.725	0.203 ± 0.013	0.187 ± 0.012	−3.936	**<0.001**
Tarsus width	0.641	0.429	0.063 ± 0.008	0.060 ± 0.004	−1.456	0.155
Foot length	2.596	0.116	0.488 ± 0.035	0.451 ± 0.023	−3.787	**0.001**
Hindlimb length	0.251	0.620	1.511 ± 0.061	1.405 ± 0.066	−4.968	**<0.001**

**Table 2:** Procrustes ANOVA testing the effects of size (log-transformed centroid size), sex, and the interaction between them on shape of appendicular skeletons of *Kaloula rugifera*, with *p* values less than 0.05 given in bold.

Skeletons	Parameters	R^2^	F	*p*
Pectoral girdle	sex	0.100	1.787	0.084
	size	0.050	1.440	0.179
	sex: size	0.023	0.825	0.468
Pelvic girdle	sex	0.122	4.729	**0.001**
	size	0.053	2.181	**0.023**
	sex: size	0.051	2.098	**0.024**
Humerus	sex	0.134	5.689	**<0.001**
	size	0.030	2.344	**0.034**
	sex: size	0.055	2.352	**0.036**
Radioulna	sex	0.058	2.432	**0.023**
	size	0.045	1.919	0.064
	sex: size	0.140	5.531	**0.001**
Femur	sex	0.205	8.757	**<0.001**
	size	0.037	1.688	0.118
	sex: size	0.060	2.726	0.023
Tibiofibula	sex	0.088	3.284	**<0.001**
	size	0.031	1.184	0.278
	sex: size	0.047	1.813	0.051
Tarsal	sex	0.124	4.822	**0.001**
	size	0.091	3.791	**0.002**
	sex: size	0.014	0.594	0.743

## Data Availability

The raw data supporting the conclusions of this article will be made available by the authors on request.

## Supplementary Materials

The following supporting information can be downloaded at https://www.mdpi.com/article/10.3390/ani15172566/s1, Figure S1: Morphospaces and deformation grids of the appendicular skeletons. Two pairs of shape meshes associated with the PC1 and PC3 represent the differences between specimens at the two ends of the corresponding shape axes (PC1 and PC3). Specifically, deformation grids showing shape change from the average (0, 0) to the negative (min) and positive (max) values of PC1 and PC3. Color corresponds to gender type, with males indicated in green, females in pink; Figure S2: Dissection of the right pectoral girdle from the ventral view of *Kaloula rugifera*. The shape of the coracoid for male and female is exhibited in the upper right corner of (a,b), respectively, highlighted in red. The superficial muscles attached to the pectoral girdle, inclding pectoralis epicoracoidea, pectoralis sternalis, and pectoralis abdominalis, which function in upper arm flexion (a,b). The deep muscles attached to the pectoral girdle, where coracobrachialis longus and coracobrachialis brevis function in pectoral girdle flexion, and coracoradialis functions in forearm flexion (c,d). Scale bar = 5 mm; Table S1: Original data of external morphology measurement, while traits of limbs are standardized to SVL.
