# Supplementary material for: Sexual Differences in Appendages of a Fossorial Narrow-Mouth Frog, Kaloula rugifera (Anura, Microhylidae)
Source: Animals (Basel). 2025 Aug 31;15(17):2566. doi: 10.3390/ani15172566 (PMC12427539; doi:10.3390/ani15172566)
Supplement: Supplementary file 1 [file animals-15-02566-s001.zip › animals-3798542-supplementary.pdf]

Sexual Differences in Appendages of a Fossorial Narrow-mouth Frog, *Kaloula rugifera* (Anura, Microhylidae)

Wenyi Zhang [1,2], Xianzheng Wang [3] , Jin Huang [3], Xiuping Wang [1,2], Bin Wang [2], Jianping Jiang [2], Bingjun Dong [1,*] and Meihua Zhang [2,*]

1 College of Life Sciences, Shenyang Normal University, Shenyang 110034, China; zhangwy0523@163.com (W.Z.); wxiuping0523@163.com (X.W.)

2 Chengdu Institute of Biology, Chinese Academy of Sciences, Chengdu 610213, China; wangbin@cib.ac.cn (B.W.); jiangjp@cib.ac.cn (J.J.)

3 Shengda Hydropower Co., Ltd., Sinohydro Group Ltd., Leshan 614000, China; scslhwangxzh@powerchina.cn (X.W.); sczssdhuangjin@powerchina.cn (J.H.)

* Correspondence: dongbingjun@synu.edu.cn (B.D.); zhangmh@cib.ac.cn (M.Z.); Tel.: +86-024-86593325 (B.D.); +86-028-82890789 (M.Z.)

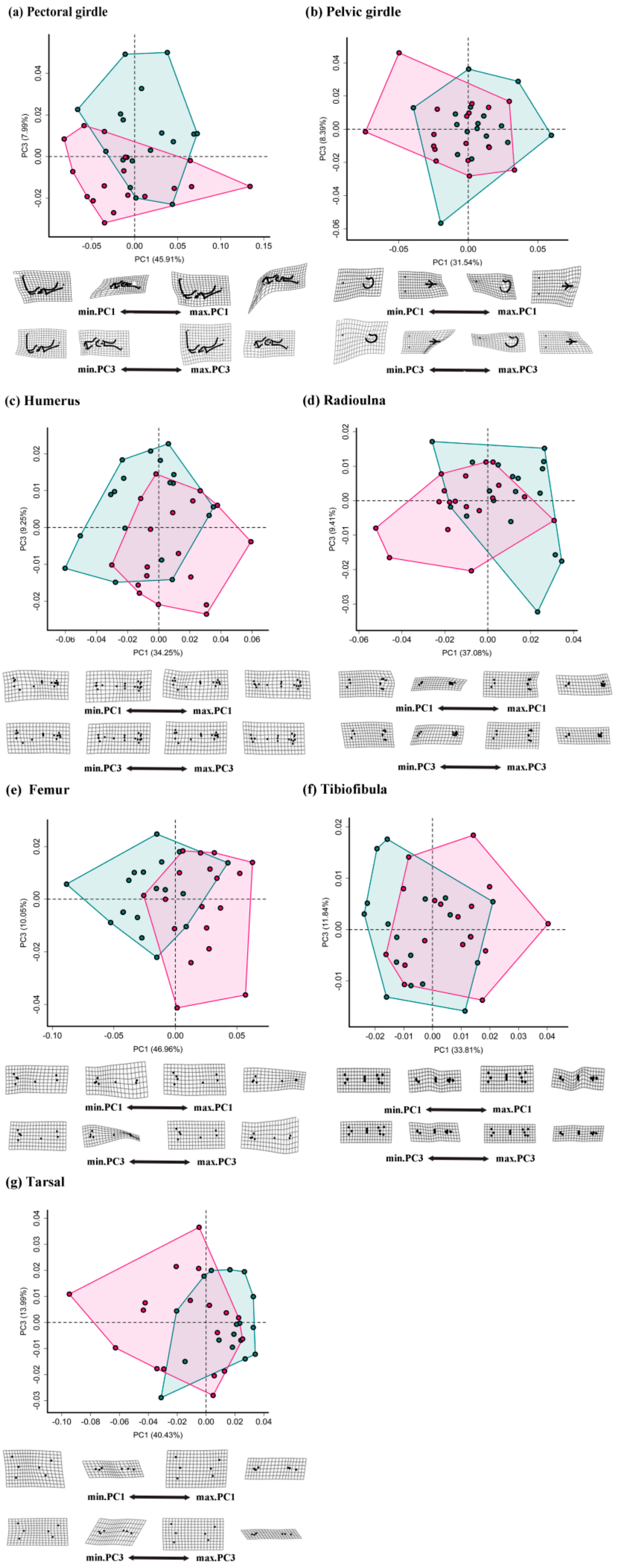
(a) Pectoral girdle
PC1 (45.91%)
PC3 (7.99%)
min.PC1
max.PC1
min.PC3
max.PC3
(b) Pelvic girdle
PC1 (31.54%)
PC3 (8.39%)
min.PC1
max.PC1
min.PC3
max.PC3
(c) Humerus
PC1 (34.25%)
PC3 (9.25%)
min.PC1
max.PC1
min.PC3
max.PC3
(d) Radioulna
PC1 (37.08%)
PC3 (9.41%)
min.PC1
max.PC1
min.PC3
max.PC3
(e) Femur
PC1 (46.96%)
PC3 (10.05%)
min.PC1
max.PC1
min.PC3
max.PC3
(f) Tibiofibula
PC1 (33.81%)
PC3 (11.84%)
min.PC1
max.PC1
min.PC3
max.PC3
(g) Tarsal
PC1 (40.43%)
PC3 (13.99%)
min.PC1
max.PC1
min.PC3
max.PC3

Figure S1. Morphospaces and deformation grids of the appendicular skeletons.Two pairs of shape meshes associated with the PC1 and PC3 represent the differences between specimens at the two ends of the corresponding shape axes (PC1 and PC3). Specifically, deformation grids showing shape change from the average (0,0) to the negative (min) and positive (max) values of PC1 and PC3. Colour corresponds to gender type, with males indicated in green, females in pink.

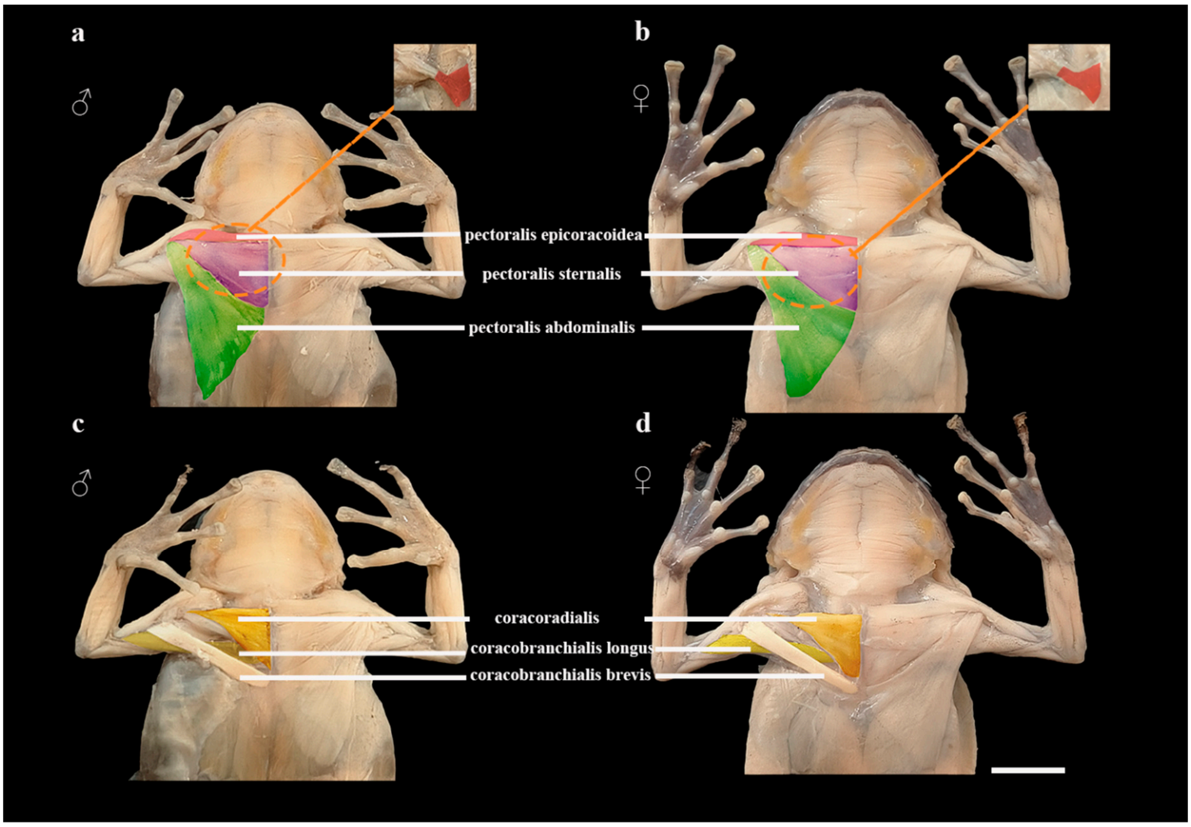

Figure S2. Dissection of the right pectoral girdle from the ventral view of *Kaloula rugifera*. The shape of the coracoid for male and female is exhibited in the upper right corner of **(a)** and **(b)** respectively, highlighted in red. The superficial muscles attached to the pectoral girdle, inclding pectoralis epicoracoidea, pectoralis sternalis, and pectoralis abdominalis, which function in upper arm flexion **(a–b)**. The deep muscles attached to the pectoral girdle, where coracobrachialis longus and coracobrachialis brevis function in pectoral girdle flexion, coracoradialis functions in forearm flexion **(c–d)**. Scale bar = 5 mm

Table S1. Original data of external morphology measurements, while traits of limbs were standardized to SVL.

Gender	Mass/ g	SVL/mm	Upper arm length	Upper arm width	Lower arm length	Lower arm width	Hand length	Forelimb length	Thigh length	Thigh width	Tibia length	Tibia width	Tarsus length	Tarsus width	Foot length	Hindlimb length
F	8.393	42.190	0.280	0.055	0.213	0.051	0.280	0.773	0.445	0.106	0.338	0.094	0.189	0.065	0.407	1.379
F	7.263	41.530	0.274	0.066	0.201	0.045	0.277	0.752	0.397	0.104	0.322	0.085	0.182	0.061	0.449	1.349
F	4.993	34.830	0.248	0.052	0.193	0.044	0.322	0.763	0.400	0.092	0.355	0.071	0.165	0.051	0.446	1.367
F	7.623	40.900	0.294	0.050	0.219	0.046	0.294	0.807	0.425	0.093	0.370	0.078	0.175	0.062	0.436	1.407
F	7.383	40.630	0.285	0.055	0.207	0.046	0.311	0.802	0.428	0.105	0.354	0.085	0.179	0.058	0.469	1.430
F	4.307	36.350	0.282	0.067	0.209	0.050	0.320	0.810	0.433	0.098	0.365	0.075	0.190	0.054	0.464	1.452
F	7.287	40.970	0.309	0.063	0.141	0.049	0.342	0.793	0.372	0.105	0.336	0.087	0.181	0.060	0.446	1.336
F	10.390	44.540	0.256	0.052	0.200	0.044	0.291	0.748	0.417	0.090	0.344	0.082	0.192	0.064	0.432	1.386
F	5.850	35.820	0.289	0.051	0.226	0.048	0.328	0.843	0.429	0.105	0.373	0.090	0.214	0.066	0.497	1.513
F	7.897	41.890	0.254	0.058	0.238	0.046	0.265	0.758	0.420	0.084	0.342	0.079	0.184	0.059	0.440	1.385
F	8.973	44.370	0.212	0.058	0.208	0.052	0.279	0.699	0.372	0.096	0.337	0.075	0.183	0.061	0.446	1.338
F	8.717	41.400	0.237	0.049	0.209	0.045	0.272	0.717	0.369	0.089	0.333	0.064	0.180	0.056	0.432	1.313
F	5.267	38.470	0.289	0.063	0.203	0.049	0.312	0.805	0.424	0.125	0.350	0.090	0.196	0.062	0.490	1.461
F	7.120	39.810	0.254	0.051	0.205	0.039	0.311	0.771	0.432	0.100	0.370	0.076	0.183	0.062	0.467	1.452
F	2.810	34.650	0.250	0.041	0.182	0.034	0.284	0.715	0.388	0.093	0.343	0.062	0.175	0.059	0.418	1.323
F	5.823	36.540	0.241	0.053	0.213	0.043	0.312	0.766	0.397	0.096	0.353	0.071	0.197	0.054	0.438	1.385
F	3.857	41.540	0.243	0.055	0.215	0.047	0.314	0.773	0.441	0.088	0.379	0.081	0.200	0.067	0.463	1.483
F	8.457	31.240	0.263	0.057	0.198	0.042	0.327	0.787	0.450	0.094	0.420	0.071	0.199	0.062	0.468	1.537
M	2.867	29.910	0.291	0.069	0.224	0.050	0.303	0.818	0.456	0.106	0.365	0.072	0.199	0.063	0.509	1.528
M	3.300	32.280	0.316	0.060	0.227	0.047	0.314	0.857	0.471	0.113	0.402	0.083	0.215	0.069	0.511	1.599
M	5.130	35.270	0.308	0.063	0.189	0.052	0.318	0.816	0.429	0.094	0.360	0.074	0.182	0.061	0.491	1.462
M	3.560	32.420	0.315	0.052	0.252	0.046	0.289	0.856	0.414	0.104	0.390	0.077	0.193	0.064	0.499	1.497

M	3.437	31.430	0.317	0.073	0.208	0.052	0.321	0.846	0.431	0.090	0.379	0.061	0.201	0.060	0.515	1.526
M	2.447	28.160	0.323	0.060	0.234	0.053	0.344	0.902	0.480	0.107	0.422	0.076	0.236	0.065	0.470	1.608
M	2.833	30.300	0.321	0.056	0.212	0.047	0.321	0.854	0.450	0.101	0.375	0.078	0.201	0.093	0.486	1.511
M	3.197	31.880	0.308	0.067	0.241	0.046	0.299	0.847	0.439	0.103	0.385	0.079	0.199	0.064	0.431	1.454
M	3.240	33.220	0.302	0.063	0.236	0.045	0.305	0.842	0.446	0.095	0.393	0.080	0.192	0.060	0.436	1.467
M	3.707	34.560	0.335	0.073	0.277	0.053	0.299	0.911	0.388	0.075	0.368	0.061	0.196	0.061	0.409	1.361
M	3.543	31.800	0.283	0.060	0.192	0.047	0.317	0.793	0.391	0.103	0.370	0.089	0.205	0.066	0.509	1.475
M	3.017	31.910	0.301	0.055	0.200	0.050	0.310	0.811	0.463	0.102	0.396	0.077	0.203	0.063	0.509	1.571
M	3.813	32.790	0.310	0.062	0.224	0.052	0.303	0.837	0.448	0.107	0.381	0.086	0.207	0.056	0.507	1.542
M	2.360	26.590	0.255	0.054	0.214	0.060	0.353	0.822	0.465	0.109	0.426	0.094	0.220	0.062	0.448	1.559
M	3.060	33.150	0.308	0.056	0.214	0.049	0.309	0.830	0.422	0.102	0.354	0.065	0.189	0.057	0.505	1.470
M	2.757	28.930	0.305	0.062	0.239	0.055	0.296	0.840	0.441	0.102	0.406	0.072	0.214	0.065	0.495	1.556
M	3.460	32.880	0.332	0.053	0.201	0.052	0.319	0.852	0.444	0.102	0.373	0.064	0.189	0.060	0.541	1.547
M	2.000	32.410	0.326	0.068	0.245	0.044	0.302	0.873	0.327	0.102	0.395	0.071	0.217	0.052	0.521	1.460
